# Supplementary material for: Recurrence of Hepatic Encephalopathy after TIPS: Effective Prophylaxis with Combination of Lactulose and Rifaximin
Source: J Clin Med. 2021 Oct 17;10(20):4763. doi: 10.3390/jcm10204763 (PMC8537523; doi:10.3390/jcm10204763)
Supplement: Supplementary file 1 [file jcm-10-04763-s001.zip › jcm-1385606-supplementary.pdf]

Table S1. Baseline characteristics by prophylactic regimen.

Parameter	None<br>% (Total Number) Or Median/Mean (sd)	LM<br>% (Total Number) Or Median/Mean (Sd)	LR<br>% (Total Number) Or Median/Mean (SD)	*p*-value
**n° of patients**	83	85	59	-
**sex**				
**male**	67.5 (56)	61.2 (52)	50.8 (30)	0.798
**female**	32.5 (27)	38.8 (33)	49.2 (29)	
**age (median, range, in y)**	58 (19-80)	57 (35-76)	59 (25-70)	0.911
**PTFE-covered stent**				
**yes**	80.7 (67)	65.9 (56)	88.1 (52)	0.944
**no**	19.3 (15)	34.1 (29)	11.9 (7)	
**HE prior TIPS**				
**yes**	9.6 (8)	11.8 (75)	50.8 (30)	-
**no**	90.4 (75)	88.2 (10)	49.2 (29)	
**HE prophylaxis**				
**none**	100 (83)	-	-	**0.002**
**RM**	-	-	-	0.298
**LM**	-	100 (85)	-	**0.008**
**LR**	-	-	100 (59)	**<0.001**
**LR+LOLA**	-	-	64.4 (38)	**<0.001**
**LRonly**	-	-	35.6 (21)	**0.046**
**etiology of liver disease**				
**alcoholic**	34.9 (29)	61.2 (52)	62.7 (37)	0.594
**viral**	12.0 (10)	10.6 (9)	1.7 (1)	
**NAFLD**	9.6 (8)	7.1 (6)	10.2 (6)	
**Other**	43.4 (36)	21.2 (18)	25.4 (15)	
**Child-Pugh grade**				
**A**	42.2 (35)	25.9 (22)	8.5 (5)	**<0.001**
**B**	54.2 (45)	70.6 (60)	57.6 (34)	
**C**	3.6 (3)	3.5 (3)	32.2 (19)	
**indication for TIPS**				
**ascites**	34.9 (29)	60.0 (51)	50.8 (30)	0.894
**variceal bleeding**	51.8 (43)	21.2 (18)	25.4 (15)	
**both**	13.3 811)	18.8 (16)	23.7 (14)	
**LTX prior TIPS**				
**yes**	9.6 (8)	3.5 (3)	-	0.319
**no**	75 (90.4)	96.5 (82)	100 (59)	
**HE after TIPS**				
**yes**	51.8 (43)	49.4 (42)	27.1 (16)	0.337
**no**	48.2 (40)	50.6 (43)	72.9 (43)	
**diabetes**				
**yes**	31.3 (26)	27.1 (23)	27.1 (16)	0.940
**no**	68.7 (57)	72.9 (62)	72.9 (43)	
**MELD-score**	13.2 (6.8)	13.3 (6.8)	19.2 (6.3)	**<0.001**
**bilirubin (mg/dl)**	1.15 (1.27)	1.10 (0.78)	2.10 (3.96)	**<0.001**
**albumin (g/dl)**	3.56 (0.76)	3.19 (0.56)	3.11 (0.51)	0.287

**creatinine (mg/dl)**	1.26 (0.79)	1.17 (0.62)	1.65 (1.19)	**0.009**
**INR**	1.21 (0.32)	1.30 (0.26)	1.53(0.41)	0.053
**platelets (ths/µl)**	133 (70)	144 (144)	128 (73)	0.892
**hemoglobin (mg/dl)**	10.7 (2.3)	10.6 (1.9)	8.6 (2.0)	**0.002**
**PSG (mmHg)**	16.7 (6.6)	17.9 (5.2)	17.5 (5.6)	**0.044**

Abbreviations: HE, hepatic encephalopathy; TIPS, transjugular intrahepatic portosystemic shunt; PTFE, polytetrafluoroethylene; NM, no prophylactic medication; LM, lactulose monoprophylaxis; RM, rifaximin monoprophylaxis; LR, lactulose and rifaximin; LOLA, l-ornithin-l-aspartate; LRonly, LR without LOLA; LR+LOLA, LR with LOLA; NAFLD, non-alcoholic fatty liver disease; LTX, liver transplantation; MELD, model of endstage liver disease; INR, international normalized ratio; PSG, portosystemic pressure gradient.

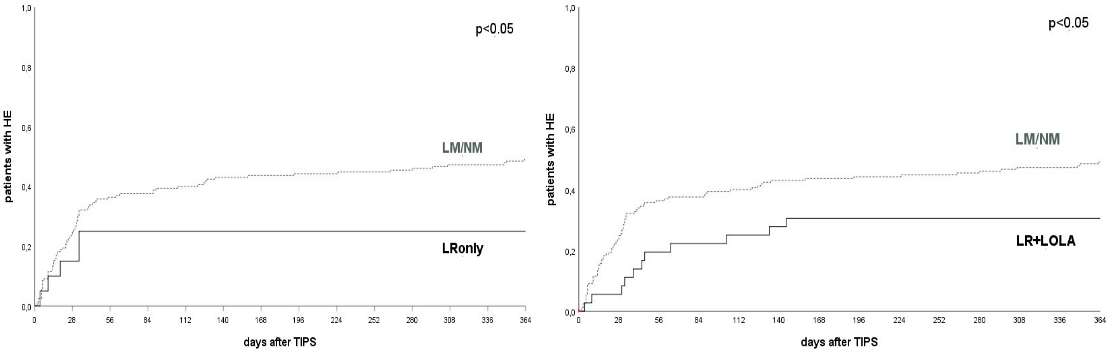

(**a**) (**b**)

Supplementary Figure S1: HE occurrence in patients with *LRonly, LR+LOLA* and *LM/NM.* (**a**) HE occurred 25% of patients with lactulose and rifaximin as HE prophylaxis and in 52.1% of patients with lactulose monoprophylaxis or no prophylactic medication (log-rank test, p<0.05). (**b**) HE occurred 29.7% of patients with lactulose, rifaximin and LOLA as HE prophylaxis and in 52.1% of patients with lactulose monoprophylaxis or no prophylactic medication (log-rank test, p<0.05). LR: lactulose and rifaximin; LM: lactulose monoprophylaxis; none: no prophylactic medication; LR+LOLA: lactulose, rifaximin and LOLA.
